# Findings of *in vitro* Analyses of Basophil Functions May Help Us Better Understand Drug Desensitization

**DOI:** 10.3389/falgy.2022.874772

**Published:** 2022-03-30

**Authors:** Masao Yamaguchi, Akiko Komiya, Maho Suzukawa, Rikiya Koketsu, Risa Shiragami, Motoyasu Iikura, Hiroyuki Nagase

**Affiliations:** ^1^Division of Respiratory Medicine, Third Department of Medicine, Teikyo University Chiba Medical Center, Chiba, Japan; ^2^Department of Clinical Laboratory, National Hospital Organization Sagamihara National Hospital, Kanagawa, Japan; ^3^Clinical Research Center, National Hospital Organization Tokyo National Hospital, Tokyo, Japan; ^4^Department of Respiratory Medicine, Nishi-Kobe Medical Center, Hyogo, Japan; ^5^Division of Respiratory Medicine, National Center for Global Health and Medicine, Tokyo, Japan; ^6^Division of Respiratory Medicine and Allergology, Department of Medicine, Teikyo University School of Medicine, Tokyo, Japan

**Keywords:** challenge test, basophil, anaphylaxis, IgE, desensitization, cell activation

## Abstract

Drug hypersensitivity can be an important problem during pharmacological management of various diseases. Patients diagnosed as having a drug allergy usually need to avoid the offending drug, either temporarily or for life. Another way of overcoming a drug allergy is to establish desensitization using the allergen drug itself. We previously investigated *in vitro* desensitization of human basophils using a subthreshold dose of an IgE-crosslinking reagent. We found that basophil desensitization occurred in a dose-dependent manner over a period of one to several hours. We think that inducible basophil desensitization occurring without histamine release may explain, at least in part, the clinical features of drug desensitization in type 1 drug allergy.

Most, if not all, clinicians have encountered adverse events during administration of drugs for diseases. Anaphylaxis, an immediate–type systemic allergic reaction, accounts for a significant portion of such adverse events (1). An IgE-mediated (type I) allergic reaction is the typical mechanism. Mast cells and basophils have numerous high–affinity IgE receptors (FcεRI) on their surface. In sensitized subjects, cross-linkage of IgE by the eliciting drug results in activation of mast cells and basophils, followed by release of their preformed mediators such as histamine, in addition to newly–synthesized mediators, including lipid mediators.

Drug-induced anaphylactic reactions have been extensively investigated from both the clinical and basic viewpoints. However, we have a poor understanding of how sensitive subjects react to exposure to very low allergen doses, i.e., below threshold. One outcome may be desensitization, but another may be worsening of the allergic reaction; the relationship between these two outcomes has been unclear.

We previously investigated basophil desensitization *in vitro* by using a simple method: human basophils were preincubated with low–dose anti-IgE antibody for various lengths of time (hours or days), and the cells were then stimulated with high–dose anti-IgE or the sensitizing allergen (2). The histamine released into the supernatant was then measured. When basophils were preincubated for 24 h with subthreshold or lower concentrations of anti-IgE antibody, the cells' shape and surface FcεRI density did not undergo significant changes, but the histamine releasability of the cells was suppressed. That suppression, i.e., desensitization, induced by anti-IgE was dose- and time-dependent. IgE-dependent histamine releasability was completely suppressed when basophils were preincubated with a near-threshold concentration of anti-IgE for 4 h. The time–course of basophil desensitization in the presence of subthreshold anti-IgE varied greatly among the tested subjects, with some being completely desensitized within only 1 h (2). Using basophils from mite-sensitive subjects, preincubation with a subthreshold allergen (Der f 2) dose induced complete unresponsiveness to later stimulation with the same allergen, and nearly complete unresponsiveness to later stimulation with high–dose anti-IgE antibody. Those findings suggest that both specific and non-specific desensitization of basophils took place (3). However, we did not perform more detailed studies to try to distinguish between specific and non-specific desensitization.

Through these analyses, we learned that *in vitro* basophil desensitization can be induced not only in the presence of high–dose allergen but also subthreshold or low–dose allergen, and that the time required for basophil desensitization by subthreshold allergen exposure varies widely from ≤ 1 h to 4 h among subjects (2, 4). Based on these results, we think the schema may reflect the features of untreated basophils (Figure 1A) and during the desensitization procedure applied to drug-sensitive patients (Figure 1B). In clinical settings, desensitization protocols usually use two-fold increments at 15-min intervals. We feel that those protocols may be optimal for safe, effective and efficient induction of basophil (and probably also mast cell) desensitization, culminating in elimination of reactivity to the drug.

**Figure 1:**
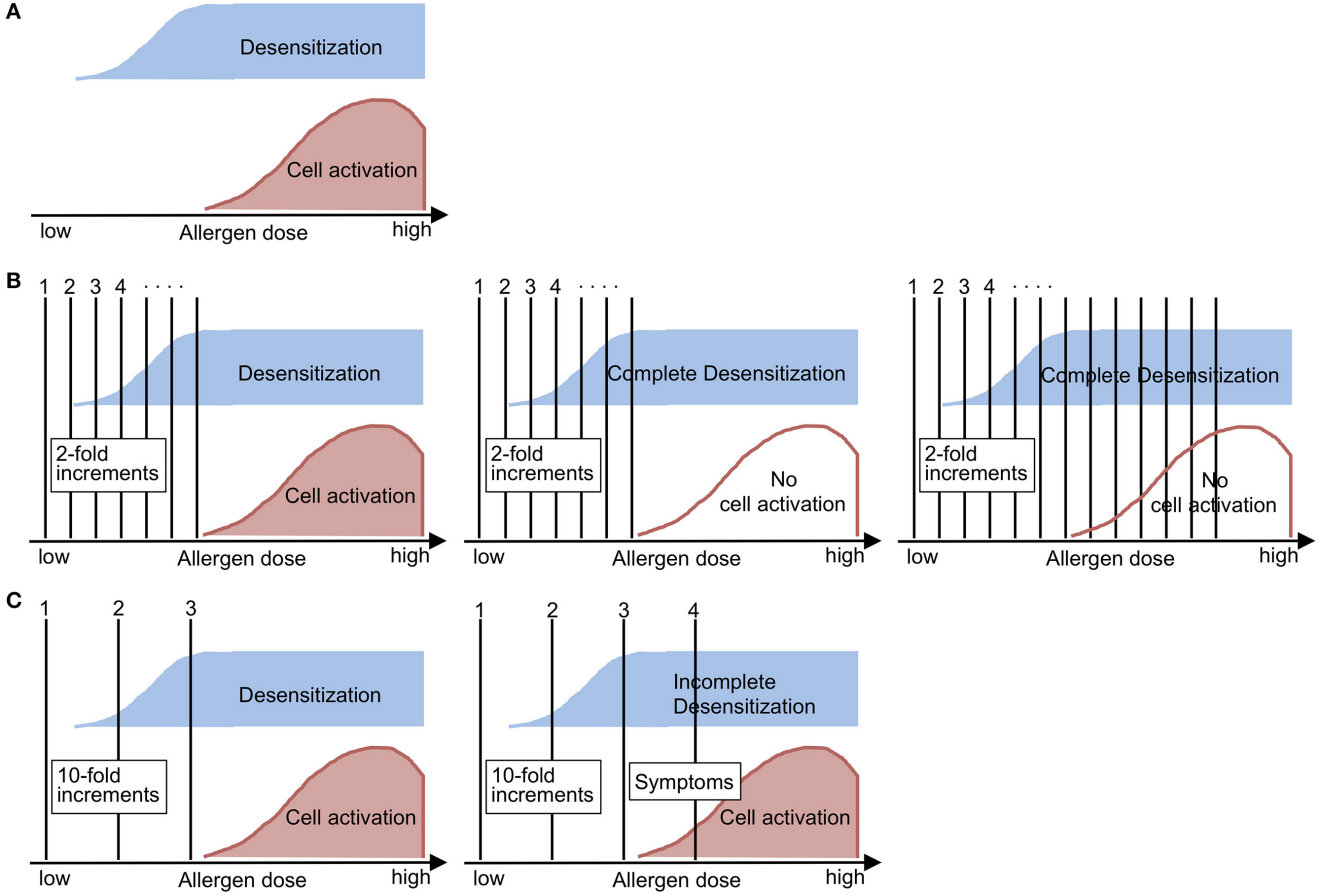
Schematic demonstrations of **(A)** the nature of allergen-induced outcomes, **(B)** desensitization and **(C)** the challenge test in sensitized subjects, as suggested by the findings of *in vitro* analysis of basophil desensitization. **(A)** Allergen-induced desensitization can occur below the lowest allergen dose that can induce cell activation (i.e., threshold dose). **(B)** During the desensitization procedure, the initial doses will induce dose-dependent desensitization (left). When complete desensitization is induced (middle), then the subject can accept incremental doses of allergen without manifesting allergic symptoms (right). **(C)** During the challenge test, the initial doses will not be able to induce complete desensitization (left), so the subject will manifest allergic symptoms when the incremental dose reaches the range of cell activation (right). This figure was translated and modified from the original figure that we published in a Japanese journal (4).

On the other hand, challenge tests utilize 10-fold or higher increments in typical settings, although the reason of those protocols has not been established. Based on the features of basophil desensitization (Figure 1A), the challenge test procedure may need to be performed rapidly enough to avoid complete desensitization during dose escalation; thus, cellular activation and either a systemic or local allergic reaction occurs when exposed to an above-threshold dose (Figure 1C). Although drug desensitization and challenge tests are performed in different situations, challenge tests can theoretically be followed by a desensitization procedure, since administration of the sensitizing drug at an above-threshold dose will accelerate desensitization and will lead to tolerance to the subsequent administration of a threshold or higher dose of the drug.

This article includes speculation and has several limitations. First, we did not perform detailed *in vivo* analyses of basophil functions during drug desensitization procedures in the clinical setting. Second, we did not test mast cell functions, but we note that precise characterization of *ex vivo* mast cell desensitization is a very difficult task. In addition, our analyses did not elucidate activation markers such as CD63 or CD203c, or the intracellular signals that induce basophil desensitization, although other researchers are accumulating findings through active scrutiny of human basophils (3, 5–8). Moreover, we do not know the extent to which desensitization induced by exposure to subthreshold allergen is similar or related to that induced by high–dose allergen. The difference between specific and non-specific desensitization is also important and needs to be investigated. Although these limitations exist, based on our daily experience we think that the relationships between desensitization and cell activation during drug dose escalation shown in Figure 1 may reflect the clinical situation.

In separate studies, we found that basophils desensitized by preincubation with subthreshold anti-IgE or anti-FcεRI antibody showed not only a suppressed response to IgE-mediated stimulation, but also enhanced responses to non-IgE-mediated stimulation, i.e., histamine release induced by Ca ionophore A23187 and chemokines, chemotaxis toward eotaxin, and expression of the CD69 surface activation marker (2, 9–11). The latter phenomenon may be out of the scope of this manuscript, and the mechanism has not yet been clarified. However, enhanced responses to non-IgE-mediated stimulation may partly explain the clinical impact of trace–level allergen exposure in patients with chronic allergic diseases, and the nature and clinical implication need to be investigated.

## Data Availability Statement

The original contributions presented in the study are included in the article/supplementary material, further inquiries can be directed to the corresponding author/s.

## Ethics Statement

The studies involving human participants were reviewed and approved by Teikyo University Ethical Review Board. The patients/participants provided their written informed consent to participate in this study.

## Author Contributions

MY, AK, MS, RK, and MI performed the *in vitro* basophil studies. MY, AK, MS, RK, MI, and HN performed the tests in clinical settings. MY and RS wrote the manuscript. All authors contributed to the article and approved the submitted version.

## Conflict of Interest

The authors declare that the research was conducted in the absence of any commercial or financial relationships that could be construed as a potential conflict of interest.

## Publisher's Note

All claims expressed in this article are solely those of the authors and do not necessarily represent those of their affiliated organizations, or those of the publisher, the editors and the reviewers. Any product that may be evaluated in this article, or claim that may be made by its manufacturer, is not guaranteed or endorsed by the publisher.
